# Bridging the nutrition divide: understanding poverty-linked inequalities in under-five children health in Pakistan

**DOI:** 10.3389/fpubh.2025.1640318

**Published:** 2025-12-05

**Authors:** Tahir Mahmood, Abidullah Khan, Nik Mohd Azim Nik Ab Malik

**Affiliations:** 1 Department of Business Administration, Sukkur IBA University, Sukkur, Sindh, Pakistan; 2 Department of Islamic Economics and Finance, Faculty of Political Sciences, Sakarya University, Serdivan, Sakarya, Türkiye; 3 Faculty of Economics and Management, Universiti Kebangsaan Malaysia, Bangi, Malaysia

**Keywords:** poverty, undernutrition, access to health, sanitation, water facilities, physical and mental health, under-five children, Pakistan

## Abstract

**Introduction:**

Despite progress in reducing stunting and underweight among children in Pakistan, significant disparities remain between poor and non-poor households. Specifically, socio-economic inequalities, limited access to healthcare, and inadequate maternal and child nutrition primarily drive these disparities. Analyzing differences in child nutritional outcomes is crucial for understanding the underlying factors contributing to health inequalities, particularly across socio-economic strata. Nevertheless, there is not enough of empirical research that specifically investigates the determinants of these disparities in Pakistan by deconstructing child nutritional inequality in the country. This study aims to address this gap by identifying the key factors contributing to unequal child nutrition outcomes.

**Methodology:**

This study examines data from the Pakistan Demographic and Health Surveys (PDHS) of 2013 and 2018, including 3,051 children from the 2013 PDHS and 4,013 from the 2018 PDHS. Height-for-age *Z* scores (HAZ) and weight-for-age *Z* scores (WAZ) are the outcome variables, alongside stunting and underweight as alternative outcome variables. Specifically Semi-parametric Method, Logistic Regression Model (LRM), and Blinder-Oaxaca decomposition are employed to investigate nutritional disparities and their fundamental drivers. The semi-parametric method identifies non-linear correlations frequently overlooked in conventional regression analysis.

**Results:**

Children from non-poor households showed significantly higher HAZ scores by 0.75 standard deviations (SDs) and WAZ scores by 0.50 SDs compared to those from poor households. Major socioeconomic factors, including maternal education, access to healthcare, sanitation, and water facilities, mainly explain this difference. Blinder–Oaxaca decomposition analysis further clarified these inequalities, indicating that 62% of the HAZ gap and 54% of the WAZ gap stem from endowment effects. The prevalence of endowment effects indicates that structural inequities, rather than behavioral differences, are the principal obstacles to attaining SDG 2.2.

**Discussion:**

This study highlights the importance of equity-focused policies to reduce child undernutrition in Pakistan by targeting key determinants such as maternal education, healthcare access, and systemic inequalities. Findings link better nutrition to improved cognitive and mental health outcomes, supporting inclusive social policies and SDG targets. Structural factors, rather than individual behaviors, primarily drive observed disparities and intergenerational health issues. Future research should explore targeted interventions addressing maternal education and service delivery inequities to achieve sustainable reductions in child undernutrition.

## 1 Introduction

According to the World Health Organization (32) report, Universal Health Coverage (UHC) requires an equitable distribution of health care for all individuals and households without financial burdens. Specifically, health equality embodies fairness in health care services, equal resource distribution, reducing disparities in health outcomes, and ultimately enhancing the welfare of all (WHO 2025). Extensive literature reveals inequalities across various indicators affecting nutritional outcomes, primarily poverty, assets, race, gender, and regional factors (2–6). For instance, disadvantaged children fall behind affluent children in significant nutritional outcomes in most developing countries. These studies emphasize the importance of addressing the nutritional gap driven by socioeconomic disparities and their subsequent policy implications (7–9). Notably, major factors contributing to the nutritional gap based on wealth and poverty status include the child's age and gender, parental education, maternal body mass index, water and sanitation facilities, and the health care system (10–15). Therefore, exploring and understanding the key drivers of the nutritional gap is essential for bridging the divide and achieving a universal, equitable healthcare system while fulfilling sustainable development goals.

Globally, undernutrition is a major public health problem; however, it is disproportionately concentrated in Africa and Asia, with the prevalence in Pakistan at higher rates. The importance of ameliorating undernutrition is well established in improving educational outcomes, enhancing labor productivity, and fostering overall economic growth and development (13–15). The latest WHO report demonstrates that stunted and underweight children represent a chronic and acute nutritional gap (WHO 2025). For instance, stunted children, indicated by low height for age, affect both their physical and mental health. Meanwhile, underweight, which is defined as low weight for age, results in chronic and severe undernutrition. The severity of undernutrition is highlighted by the recently released global statistics. For example, nearly 150 million children are stunted, while 37 million are underweight. Their prevalence is significantly higher in Africa and South Asia, which accounts for a staggering 90 percent of the overall global burden of undernutrition (17). These alarming statistics indicate that despite some achievements, it remains exceedingly challenging to meet the targets set by the SDGs 2030.

Upon examining the available dataset, the pattern of undernutrition in Pakistan reveals that the prevalence of stunted children was 45% in 2013, declining to 38% in 2018. Similarly, the prevalence of underweight children decreased from 27 to 23% during the same period. Nevertheless, this prevalence of stunting remains the highest in the region. This elevated rate of undernutrition poses significant challenges to child survival, mental development, and overall economic growth and productivity. To ward off this problem, the government has implemented numerous health policies and intervention programs. For example, the National Nutrition Policy 2018 focuses on improving mothers and children, multisectoral nutrition strategies, BISP, micronutrient supplementation, and scaling up nutrition (SUN) movements. Despite efforts, progress remains uncertain in achieving the targets aimed at reducing stunting by 3.9% per year by 2025 from the 2010 baseline (1).

The causes of undernutrition in Pakistan are complex, stemming from various socioeconomic, political, and environmental factors that impede health, human capital, and economic growth (2). Specifically, numerous studies in Pakistan have investigated the prevalence of malnutrition. For instance, studies conducted by Bhutta et al. (3), Zaidi et al. (4), and Soofi et al. (2) link undernutrition to poverty, food insecurity, and social and economic factors (5, 6). Likewise, Asif and Akbar (7) examine trends in socio-economic inequalities related to child undernutrition, analyzing causes through decomposition using 2017–18 Pakistan Demographic and Health Surveys (PDHS) data. The study highlights significant disparities in education, with parental education and wealth being key factors. It recommends targeted policies for both rural and urban areas and suggests that fair wealth distribution could help reduce rural-urban gaps. Tariq et al. (8) research identifies socio-demographic, nutritional, and health factors associated with stunting, wasting, and underweight in children under two, emphasizing child age, BMI, access to information, birth order, parental education, rural residence, and sanitation. It advocates tackling poverty, low parental education, micronutrient deficiencies, and focusing on provinces with high rates of undernutrition. Raju and Dsouza (9) provide a narrative review of existing literature on child undernutrition, discussing food consumption, regional differences, determinants, and intervention effects. Ali (10) highlights factors such as poverty, food insecurity, maternal health, age at marriage, and education, alongside low birthweight, prematurity, breastfeeding practices, diet, health, and socioeconomic and cultural influences affecting early childhood growth. Furthermore, malnutrition adversely affects children's health, survival, and cognitive development. The findings confirm that children born with intervals longer than three years are less likely to be stunted or underweight, and wealthier households tend to have lower rates of stunting and wasting. These insights can inform the integration of nutrition policies with children's education, maternal healthcare, and social protection programs for resource-poor families, aiming to reduce undernutrition among children under five (11, 12).

While it is true that a few studies have examined undernutrition among under-five children in Pakistan, this study offers several distinct contributions that extend the existing literature. First, it classifies households into two groups, poor and non-poor, based on an asset index, enabling a nuanced comparison of undernutrition across socioeconomic strata. Second, it combines two PDHS waves (33, 34), thereby increasing the sample size and enhancing the robustness of the findings. Moreover, by incorporating year dummies within a pooled regression framework, the study controls for potential policy and contextual changes between the two periods, an approach seldom employed in previous research.

Undernutrition varies systematically by socio-economic status and is unevenly spread across regions. Classifying households as poor or non-poor helps measure the nutritional outcome gap related to poverty. This supports social gradient theory, which indicates that health and nutrition outcomes worsen as socio-economic position declines. Additionally, the Blinder–Oaxaca decomposition requires two groups to estimate how much of the nutrition gap is explained by observable factors, like education, access to healthcare, and sanitation, vs. unobserved structural factors. From a policy view, nutrition programs are more successful when tailored to the specific needs of vulnerable groups. Therefore, this analysis guides policymakers in creating targeted social protection and health interventions. Empirically, poverty acts as a confounder when examining the relationship between nutrition and factors such as education, water and sanitation, and healthcare access. Comparing poor and non-poor groups enables better control for income effects and reduces bias in estimating the influence of non-income factors.

Building on the previous discussion, the study sets out clear objectives: first, to quantify the nutritional gap caused by poverty by comparing undernutrition between poor and non-poor households across regions. Second, to decompose this nutrition gap into contributions from measurable socio-economic and demographic factors vs. unobserved structural factors. Lastly, to inform targeted policy by identifying poverty-specific barriers impacting child nutrition outcomes.

**Figure 1:**
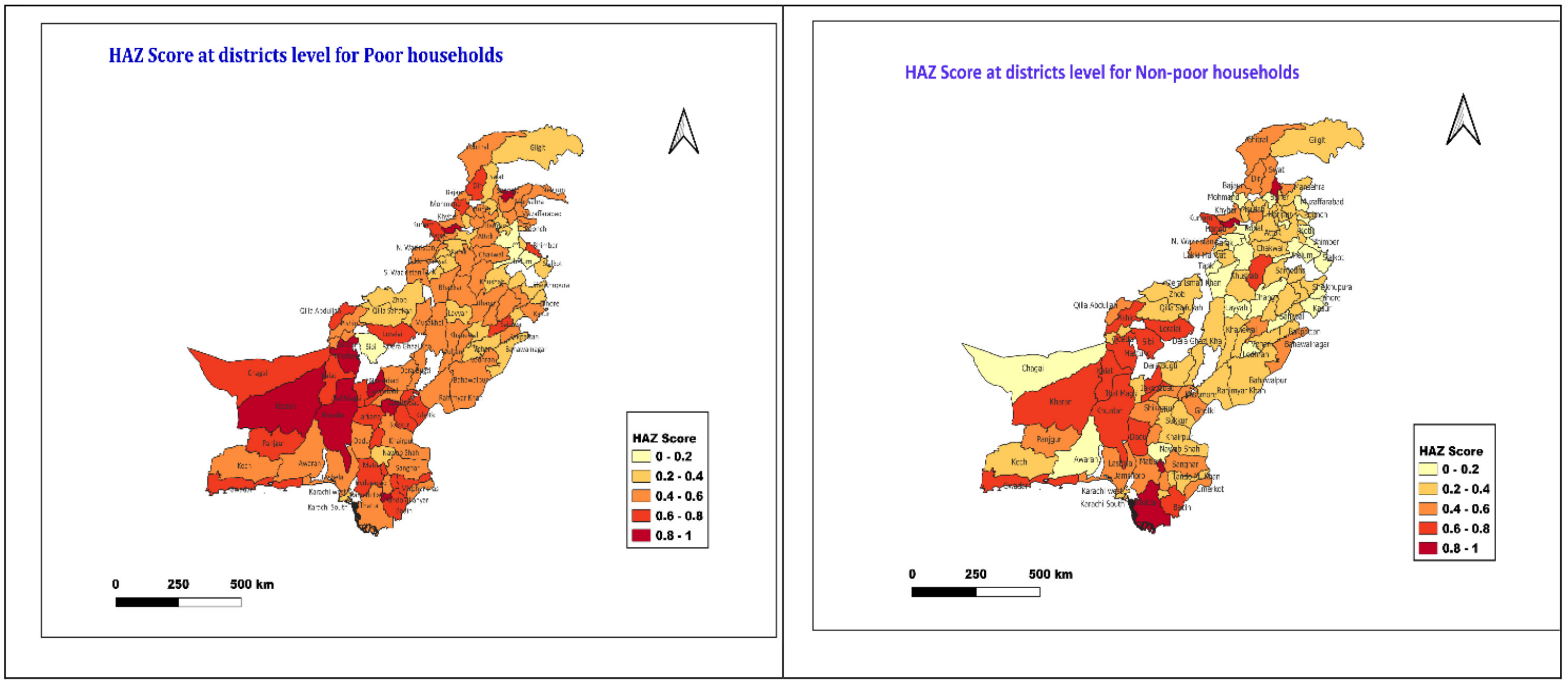
Map of Pakistan—district-wise HAZ scores for the poor and non-poor groups.

In this regard, the Blinder–Oaxaca decomposition technique is used to meet these objectives (13). The rationale of this study is that the evidence obtained from the specific objectives will assist policymakers and planners in Pakistan in designing targeted interventions for poor households, thereby contributing to the achievement of the SDG of reducing inequality by 2030, alongside the targets of eliminating all forms of malnutrition and achieving UHC. Figure 1 shows the district-wise HAZ scores for the poor and non-poor groups. The map of Pakistan was created via QGIS 3.34 while the shapefile was accessed from a publicly available source.

This paper is structured as follows: Section 2 provides an overview of the data sources and the methodological framework. Section 3 presents empirical findings, while Section 4 offers an in-depth discussion of the results.

## 2 Materials and methods

### 2.1 Data source and sampling design

This study is based on the 2013 and 2018 rounds of the PDHS, implemented by the National Institute of Population Studies (NIPS) under the aegis of the Ministry of National Health Services, Regulations, and Coordination. The research utilized sampling weights from the PDHS to guarantee national representation and considered survey design effects in all analyses. This dataset provides a comprehensive overview of population, maternal, and child health issues in Pakistan. It employs a stratified two-stage sample design. Stratification is performed by separating the provinces into urban and rural domains. Independent samples are selected in every stratum through a two-stage selection process. The first stage involved selecting clusters consisting of enumeration blocks or villages. The cluster villages are drawn with a probability proportional to their size, which is the number of households residing in the village at the time of the census. The survey was carried out in a total of 498 areas for the years 2012–13. A total of 580 clusters were selected during the 2017–18 data collection. The second stage consisted of a systematic sampling of households. A household listing operation was undertaken in all the selected clusters, and a fixed number of 28 households per cluster was selected with an equal probability systematic selection process, for total sample sizes of approximately 14,000 and 16,240 households in 2013 and 2018, respectively. The dataset includes data on household and individual socioeconomic and demographic characteristics. Further details about the survey design are available elsewhere (14).

### 2.2 Study population

After excluding children with missing anthropometric or socioeconomic data, the final analytic sample comprised 3,051 children (33) and 4,013 (34). Households were classified as poor (bottom 40%) and non-poor (top 60%) based on asset index scores, following Joe et al. (15). This approach captures relative inequality within the national context. The data are collected by interviewing mothers of children aged 15–49 years. In addition, missing information is assumed to be random (16).

### 2.3 Outcome and decomposition variables

The HAZ and WAZ of children under 5 years of age (0–59 months), measured on a continuous numeric scale, are selected as the outcome variables. Specifically, the height-for-age provides a measure of children's height relative to their age, demonstrating cumulative linear growth. It responds to long-term nutritional deprivation and chronic or frequent illnesses. Conversely, the body weight of children is sensitive to short-term variations in nutrition and illness (17). As a result, HAZ is more commonly used as an indicator of child nutrition or chronic undernutrition (18). It is calculated by dividing the difference between a child's height and the median value for the reference population for the corresponding age and sex by the standard deviation (SD) of the reference population (19). Notably, the PDHS adopts the WHO growth standards as the reference population. Importantly, the study specifies stunting and underweight as alternative outcome indicators along with HAZ and WAZ, in line with Child Growth Standards (20). For example, stunting, which is defined as a HAZ below 2 standard deviations (SD) from the WHO reference median, indicates chronic undernutrition and long-term essential nutrient deficiencies. Underweight, which is defined as a WAZ below 2 SD, reflects a composite measure of both acute and chronic undernutrition. Employing binary classifications as an alternative model specification, coded as 1 if the child is stunted or underweight and 0 otherwise, we used the LRM to examine the consistency of socioeconomic and maternal determinants of undernutrition. This approach complements the *Z*-score analysis and aligns the findings with policy-relevant cutoffs, as suggested by WHO. The alternative model specification may enhance the practical interpretability and policy relevance of the results.

Moreover, the decomposition variable is constructed via the asset index, which is derived via principal component analysis (PCA). The asset index is generated based on binary indicators, including access to electricity, ownership of a television, motorcycle, landline telephone, mobile phone, IT facilities, use of a clean cooking facility, and the materials used for flooring and roofing. The index is rescaled to range from a minimum score of 0 to a maximum score of 10. Households are then classified into poor and non-poor groups based on their asset index scores. The bottom 40% of households are categorized as poor, whereas the top 60% are classified as *non-poor*. This categorization is in line with the literature (15, 21, 22). Importantly, the asset index is widely used as a robust indicator to measure the living standards of households (15). A description of the outcome variables and the decomposition variable is given in Table 1.

**Table 1:** Descriptive statistics of outcome and independent variables.

**Variables**	***N***	**Mean/Proportion**	**SD**	**Min**	**P50**	**Max**
HAZ-Score	7,064	−1.658	1.770	−6	−1.64	5.81
WAZ-Score	7,064	−1.154	1.293	−5.86	−1.12	4.26
Stunting	2,914	0.412	0.492	0	–	1
Underweight	1,689	0.239	0.426	0	–	1
Decomposing var-poor	3,320	0.469	0.499	0	–	1
Asset index (0–10)	7,064	4.391	2.607	0	4.272	10
Gender-female	3,496	0.494	0.5000	0		1
Child age (continuous)	7,064	29.658	17.303	0	30	59
Child age month (0–11)	1,369	0.193	0.395	0	–	1
Month (12-23)	1,346	0.190	0.392	0	–	1
Month (24-35)	1,447	0.204	0.403	0	–	1
Month (36–47)	1,443	0.204	0.403	0	–	1
Month (48–59)	1,459	0.206	0.404	0	–	1
Small size at birth	1,213	0.171	0.377	0	–	1
Diarrhea prevalence	1,447	0.204	0.403	0	–	1
Prenatal checkups	4,607	0.652	0.476	0	–	1
Hospital delivery	4,276	0.605	0.488	0	–	1
Cesarean delivery	1,154	0.163	0.369	0	–	1
Postnatal check ups	1,617	0.228	0.420	0	–	1
Birth order	7,064	3.367	2.292	1	3	15
Mother's age (years)	7,064	29.229	6.101	15	29	49
Mother height (cm)	7,064	154.836	5.877	106.7	154.8	196.6
Maternal education	7,064	4.238	5.133	0	0	16
Husband education	7,064	6.786	5.226	0	8	16
Mother smoking	276	0.039	0.194	0	–	1
Household size	7,064	9.407	4.986	2	8	48
BISP	321	0.045	0.2082	0	–	1
No water facility	719	0.101	0.302	0	–	1
No toilet facility	1,042	0.147	0.354	0	–	1
Provinces-Punjab	1,875	0.265	0.441	0	–	1
Sindh	1,458	0.206	0.404	0	–	1
KP	1,208	0.171	0.376	0	–	1
Baluchistan	752	0.106	0.308	0	–	1
GB	575	0.081	0.273	0	–	1
ICT	437	0.061	0.240	0	–	1
AJK	427	0.060	0.230	0	–	1
FATA	332	0.046	0.211	0	–	1
Region-rural	3,147	0.445	0.497	0	–	1
Year-2018	4,013	0.568	0.495	0	–	1

### 2.4 Independent variables

The study employs a comprehensive set of independent variables: child's age (in months), gender, size at birth, diarrhea incidence, prenatal checkups, hospital and cesarean deliveries, postnatal checkups, birth order, mother's age, maternal height, maternal and paternal education levels, mother smoking, household size, participation in the Benazir Income Support Program (BISP), access to clean drinking water and sanitation facilities, provinces including the capital territory, FATA, AJK, GB, and regional strata. The child's age in completed months at the time of the survey is categorized into five groups. The child's gender refers to the biological sex reported by the mother. The mother's subjective assessment of the child's size at birth is categorized as very large, larger than average, smaller than average, and very small; the last two are considered small at birth. Diarrhea prevalence is defined as whether the child experienced diarrhea in the 2 weeks preceding the survey. Prenatal checkups, hospital delivery, cesarean delivery, and postnatal checkups are considered binary variables (no/yes). Birth order indicates the child's position among all live births to the mother (e.g., 1st child, 2nd child). The mother's age, height, and maternal and paternal education levels are treated as numeric variables. The BISP is coded as a binary variable indicating whether the household is a beneficiary of BISP cash transfer assistance (no/yes). Accessibility to drinking water is classified according to the WHO as either improved or unimproved. Access to sanitation facilities is similarly classified as a binary variable indicating whether the household has access to an improved sanitation facility.

### 2.5 Method of analysis

This study uses both non-parametric and parametric econometric techniques to investigate the nutritional outcome for non-poor and poor households. The non-parametric regression explores non-linear relationships, logistic regression identifies determinants, and the Blinder–Oaxaca decomposition quantifies non-poor and poor group differences. In this regard, Kernel Density is used to visualize the distribution of the data, gaining meaningful insights between two comparison groups. Moreover, the study assesses the associations between nutritional outcomes *(Y)* for child *i* at time *t* and a vector of time-varying determinants *(X)* and trend effects represented by a vector of year dummy variables *(T)* to control for any unobserved time-varying national factors that might influence nutrition outcomes. The analysis used both Ordinary Least Squares (OLS) for *Z*-Score as outcome variable and LRM for stunting and underweight as outcome variables.

This association is represented in equation form as given below.


$$\begin{array}{c} Y_{i,t\;}=\;\beta X_{i,t}+T+U_{i,t} \end{array}$$

The vector of coefficients (β) and the set of parameters to be estimated are denoted by X, including white noise.

The Blinder–Oaxaca decomposition method (13, 23) is used to determine the factors that contributed to the mean difference in nutritional outcomes between the non-poor and poor groups. Specifically, the nutrition outcome represented by *Y* as a linear function of observable characteristics *X*, with separate equations for poor (*P*) and non-poor (*NP*) households:


$$\begin{array}{ccc} Y_{P} & = & \beta_{P}X_{P}+\varepsilon_{P}\\ Y_{NP} & = & \beta_{NP}X_{NP}+\varepsilon_{NP} \end{array}$$

The mean outcomes for the two groups are as follows:


$$\begin{array}{ccc} {Ȳ}_{P} & = & \beta_{P}{\bar{X}}_{P}\\ {Ȳ}_{NP} & = & \beta_{NP}{\bar{X}}_{NP} \end{array}$$

The difference in mean outcomes between non-poor and poor individuals is as follows:


$$\begin{array}{c} Y=\;{Ȳ}_{NP}-\;{Ȳ}_{P} \end{array}$$

The Oaxaca decomposition splits Δ*Y* into two components:


$$\begin{array}{c} E=\left({\bar{X}}_{NP}-\;{\bar{X}}_{P}\;\right)\beta^{*} \end{array}$$

This reflects differences in observable characteristics, such as differences in mothers' education, antenatal care checkups, etc., between non-poor and poor households. This component is commonly known in the literature as the explained component-endowment effect.


$$\begin{array}{c} U=\left(\beta_{NP}-\;{\bar{\beta }}_{P}\;\right)X^{*} \end{array}$$

This reflects differences in coefficients that represent structural or unobservable factors such as discrimination or differences in return or unequal access to opportunities. This component is commonly called an unexplained component—the coefficient effect.

The explained and unexplained components can be combined, excluding the intercept and interaction terms.


$$\begin{array}{c} Y=\left[\left({\bar{X}}_{NP}-\;{\bar{X}}_{P}\right)\beta^{*}\right]+\left[\left(\beta_{NP}-\;{\bar{\beta }}_{P}\;\right)X*\right] \end{array}$$

This decomposition helps policymakers understand whether poverty differences arise from disparitiexs in endowments or unequal returns to those endowments. The Blinder–Oaxaca decomposition method adopts a counterfactual approach that involves replacing the coefficients and variable levels of one group with the corresponding values of another group (reference group). In this analysis, we specified the non-poor group as a reference group to obtain the expected change in the predicted mean outcome when the poor group received the predictor values and regression coefficients from the non-poor group.

## 3 Empirical results

Table 1 presents the descriptive statistics (mean, proportion, SD, Min and Max) of the outcome and independent variables. The dummy and categorical variables are represented by their proportion (percent), while continuous variables are illustrated by mean values. The mean HAZ score and WAZ score are −1.65 and −1.15, respectively, indicating substantial growth deficits. Specifically, 41% of children are classified as stunted and 23% as underweight, according to WHO standard thresholds. The asset index–based classification indicates that 46% of households fall into the “poor” group, while the remaining 54% are classified as “non-poor.”

About 49% of the sample comprises female children. Children have an average age of 29 months. The analysis further reveals that 17% (0.17) of the children were reported as small at birth and 20% suffered from diarrhea and 60% of deliveries occurred in hospitals, with 16% through cesarean section. While 65% of mothers had prenatal checkups, only 22% had postnatal checkups. Mothers have an average age of 29 years and an average height of 155 cm. Furthermore, the average years of schooling are four for mothers and seven for their spouses. While, maternal smoking prevalence stands at 3%. The average household size consisted of nine members and only 4% of the households received BISP support. Furthermore, 10% of the households lacked access to clean drinking water, and 14% had inadequate sanitation. Geographically, 26% of the sample came from Punjab, 20% from Sindh, and 17% from Khyber Pakhtunkhwa, with 44% from rural areas. The PDHS 2018 represented 57% of the sample, while the remaining 43% was from the PDHS 2013. Finally, the Variance Inflation Factors (VIF) indicated no evidence of multicollinearity among the variables.

**Figure 2:**
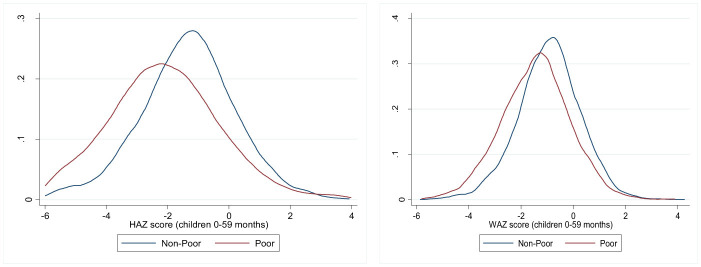
Shifts in the distribution of HAZs and WAZ scores across non-poor and poor households. Source: Author's estimation of Kemel density estimates from the PDHS-2013-18.

Figure 2 shows the kernel density estimations (KDE) of the distributions of child HAZ and WAZ for both non-poor and poor households. This demonstrates an almost unequal shift in the entire distribution of the HAZ. Specifically, the KDE curve for non-poor households is shifted to the right compared with that for poor households, which indicates that children in wealthier families have higher average nutritional scores. In contrast, a wider KDE curve for a poor group suggests greater variability in nutritional status within the poor group. Moreover, an almost similar pattern, although with less variation, is observed for the WAZ, as shown in Figure 2.

**Figure 3:**
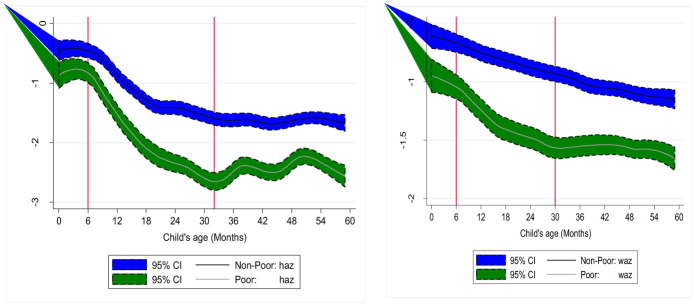
Shifts in HAZ and WAZ scores by child age for non-poor and poor households. Source: Author's calculation of local polynomial smoothing predictions with 95% CIs.

Figure 3 shows the predicted HAZ and WAZ by child age, revealing a non-linear relationship between these variables. For example, considering stunting, the curve illustrates two pivotal dimensions: the influence of maternal nutrition on birth size and the postnatal growth trajectory. The intercept reflects differences in child size at birth, underscoring maternal malnutrition as a key constraint, which is particularly evident in the significant disparity in birth size and prenatal checkups between poor and non-poor households. Postnatal growth faltering is most pronounced from 6 to 30 months of age, aligning with the transition to solid foods, liquids beyond breast milk, and increased mobility that elevates disease exposure. During this period, the HAZ gap between poor and non-poor households steadily widened, indicating socioeconomic disparities in dietary diversity and health. These findings underscore the importance of minimum dietary diversity interventions and provide evidence to guide policy by identifying age-specific thresholds where interventions can have the greatest impact. A nearly parallel but somewhat linear association was exhibited by WAZ.

Table 2 presents disparities in outcome variables, specifically HAZ and WAZ, as well as key explanatory variables between poor and non-poor households. On average, non-poor households present significantly higher HAZ and WAZ scores by 0.75 standard deviations (SD) and 0.50 SD, respectively, than poor households do. Similarly, stunting prevalence is 31% among non-poor households and 52% among poor households, while underweight prevalence is 16 and 31%, respectively, reflecting a statistically significant disparity of 0.21 and 0.15. Among the key explanatory variables, significant differences were observed between the two income groups in determinants such as maternal and paternal education levels, access to basic healthcare services (prenatal checkups, hospital deliveries, cesarean deliveries, and postnatal checkups), birth order, availability of water and sanitation facilities, and provincial and regional domains. For instance, the mean maternal education score was notably lower among poor households (1.7) than among non-poor households (6.4), reflecting a significant disparity of 4.7 points. Similarly, the mean paternal education score for poor households was 4.7, whereas it was 8.6 for non-poor households, indicating a substantial difference of 3.9 points between the two groups.

**Table 2:** Differences in the mean values of the outcome and explanatory variables between non-poor and poor households.

**Household categories**	**Mean HAZ score**	**Mean WAZ score**	**Stunting**	**Underweight**	**Small size at birth**
Non-poor	−1.307	−0.917	0.313	0.169	0.158
Poor	−2.056	−1.423	0.524	0.317	0.188
Difference	0.749^***^	0.506^***^	−0.211^***^	−0.148^***^	−0.03^**^
	**Diarrhea**	**Household head**	**Mother age**	**Mother height**	**Mother education**
Non-poor	0.195	0.090	29.225	155.295	6.473
Poor	0.216	0.087	29.234	154.319	1.719
Difference	−0.021^*^	0.003	−0.080	1	4.754^***^
25.8-1.55,16.5	**Paternal education**	**Mother smoke**	**BISP**	**Pre Natal**	**Delivery hospital**
Non-poor	8.627	0.033	0.033	0.752	0.755
Poor	4.711	0.046	0.060	0.539	0.437
Difference	3.916^***^	−0.013	−0.027^**^	0.213^***^	0.318^***^
25.8-1.55,16.5	**Cesarean delivery**	**Postnatal**	**Birth order**	**Water facility**	**Toilet facility**
Non-Poor	0.237	0.262	2.990	0.058	0.024
Poor	0.080	0.191	3.794	0.152	0.287
Difference	0.156^***^	0.071^***^	−0.804^*^	−0.094^**^	−0.263^***^
16.8-1.55,16.5	**Household size**	**Sindh**	**KP**	**Baluchistan**	**GB**
Non-poor	9.329	0.198	0.159	0.088	0.067
Poor	9.497	0.216	0.184	0.127	0.098
Difference	−0.168	−0.018	−0.025	−0.039^**^	−0.031^**^
16.8-1.55,16.5	**ICT**	**AJK**	**FATA**	**Region**	
Non-poor	0.101	0.082	0.013	0.647	
Poor	0.018	0.036	0.085	0.219	
Difference	0.082^***^	0.045^***^	−0.072^***^	0.428^***^	

^***^p < 0.01, ^**^p < 0.05, ^*^p < 0.1.

Significant disparities in access to basic health services were observed between poor and non-poor households. Prenatal care coverage was 75% for non-poor households and 53% for poor households, reflecting a 21% difference. Hospital delivery rates showed a 31% gap, with 75% for non-poor households and 43% for poor households. The cesarean delivery rates were 23% for non-poor households and 8% for poor households, a difference of 15%. Postnatal care coverage was 26% for non-poor households and 19% for poor households, showing a 7% disparity. Additionally, water facility access was 9% lower, and sanitation facility access was 26% lower for poor households. Compared with that among urban areas, rural residency among poor households was 46% greater.

Table 3 presents the OLS and LRM estimates for the determinants of child growth outcomes, with HAZ and WAZ representing continuous measures, and stunting and underweight as their respective binary indicators. Columns 2 and 3 report the regression coefficients for HAZ and WAZ, respectively. In columns 4 and 5, the estimates of LRM are given. The findings reveal several significant associations.

**Table 3:** Determinants of child growth in pooled regression models.

**Outcome variable**	**HAZ score**	**WAZ Score**	**Stunting**	**Underweight**
**Method**	**OLS**	**OLS**	**LRM**	**LRM**
Asset index (1-10)	0.064^***^	0.045^***^	−0.101^***^	−0.084^***^
	(0.011)	(0.008)	(0.016)	(0.018)
**Child sex (reference category-male)**				
Female child	0.076^**^	0.025	−0.133^**^	−0.142^**^
	(0.037)	(0.028)	(0.054)	(0.060)
**Child age (reference category-0–11)**				
Category-12–23 months	−0.934^***^	−0.302^***^	1.044^***^	0.190^*^
	(0.060)	(0.045)	(0.094)	(0.101)
Category-24–35 months	−1.384^***^	−0.449^***^	1.587^***^	0.465^***^
	(0.060)	(0.045)	(0.094)	(0.099)
Category-36–47 months	−1.441^***^	−0.561^***^	1.581^***^	0.505^***^
	(0.062)	(0.047)	(0.097)	(0.103)
Category-48–59 months	−1.365^***^	−0.607^***^	1.429^***^	0.520^***^
	(0.064)	(0.048)	(0.100)	(0.107)
**Birth size (reference category-large)**				
Small size	−0.328^***^	−0.383^***^	0.420^***^	0.516^***^
	(0.049)	(0.037)	(0.071)	(0.075)
**Had diarrhea (reference category-no)**				
Yes	−0.128^***^	−0.165^***^	0.132^*^	0.257^***^
	(0.047)	(0.035)	(0.068)	(0.073)
**Prenatal check (reference category-**<**4)**				
>4	0.072	0.111^***^	−0.090	−0.122^*^
	(0.044)	(0.033)	(0.064)	(0.069)
**Hospital delivery (reference category-No)**				
Yes	0.155^***^	0.099^***^	−0.154^**^	−0.174^**^
	(0.045)	(0.033)	(0.063)	(0.069)
**Delivery cesarean (reference category-No)**				
Yes	0.179^***^	0.099^***^	−0.205^**^	−0.425^***^
	(0.056)	(0.033)	(0.085)	(0.104)
**Postnatal check (reference category-No)**				
Yes	−0.033	0.099^***^	0.073	0.030
	(0.048)	(0.033)	(0.071)	(0.079)
Number of birth order	−0.037^***^	−0.001	0.038^**^	−0.003
	(0.013)	(0.009)	(0.018)	(0.020)
Woman's age in years	0.025^***^	0.011^***^	−0.026^***^	−0.015^**^
	(0.005)	(0.003)	(0.007)	(0.007)
Respondent's height in centimeters	0.050^***^	0.031^***^	−0.066^***^	−0.042^***^
	(0.003)	(0.002)	(0.005)	(0.005)
Maternal education	0.018^***^	0.021^***^	−0.032^***^	−0.040^***^
	(0.005)	(0.004)	(0.008)	(0.009)
Paternal education	0.009^**^	0.008^**^	−0.015^**^	−0.013^*^
	(0.004)	(0.003)	(0.006)	(0.007)
**Mother smokes (reference category-No)**				
Yes	−0.471^***^	0.201^***^	0.466^***^	−0.333^**^
	(0.098)	(0.074)	(0.145)	(0.152)
**Water facility (reference category-Yes)**				
No	−0.054	−0.085^*^	−0.083	0.134
	(0.066)	(0.049)	(0.094)	(0.099)
**Toilet facility (reference category-Yes)**				
No	−0.100^*^	−0.031	0.096	0.020
	(0.059)	(0.044)	(0.083)	(0.086)
Household size	−0.011^***^	−0.003	0.012^**^	0.006
	(0.004)	(0.003)	(0.005)	(0.006)
**BISP (reference category-No)**				
Yes	0.024	−0.109	−0.089	0.148
	(0.094)	(0.070)	(0.131)	(0.142)
**Provinces (reference category-Punjab)**				
Category-Sindh	−0.536^***^	−0.495^***^	0.653^***^	0.842^***^
	(0.056)	(0.042)	(0.080)	(0.087)
Category-KP	0.119^**^	0.167^***^	0.023	−0.045
	(0.059)	(0.045)	(0.086)	(0.099)
Category-Baluchistan	−0.630^***^	−0.298^***^	0.755^***^	0.612^***^
	(0.075)	(0.056)	(0.107)	(0.112)
Category-GB	0.069	0.589^***^	0.174	−0.761^***^
	(0.076)	(0.057)	(0.109)	(0.148)
Category-ICT	0.171^**^	0.079	−0.148	−0.188
	(0.084)	(0.063)	(0.134)	(0.168)
Category-AJK	−0.090	0.046	0.114	−0.148
	(0.088)	(0.066)	(0.135)	(0.166)
Category-FATA	−0.471^***^	0.062	0.601^***^	−0.023
	(0.101)	(0.076)	(0.141)	(0.158)
**Region (reference category-rural)**				
Urban	0.033	0.023	0.033	−0.039
	(0.044)	(0.033)	(0.064)	(0.073)
**Year (reference category-2013)**				
Year = 2018	0.065	0.057^*^	−0.111^*^	−0.101
	(0.044)	(0.033)	(0.064)	(0.070)
Constant	−9.239^***^	−6.223^***^	9.760^***^	5.980^***^
	(0.505)	(0.378)	(0.775)	(0.819)
Observations	7,064	7,064	7,064	7,064
R-squared/Pseudo *R*^2^	0.240	0.201	0.145	0.110

Standard errors in parentheses.

^***^p < 0.01, ^**^p < 0.05, ^*^p < 0.1.

Specifically, a one-unit increase in the 1–10-scale asset index is associated with a 0.06 standard deviation (SD) improvement in HAZs and a 0.04 SD improvement in WAZs. This implies a notable socioeconomic gradient, where children from the richest households exhibit a 0.60 SD advantage in growth outcomes compared with those from the poorest households. Compared with male children, female children demonstrated a 0.067 SD improvement in HAZs, although no significant gender effect was observed for WAZs. Both the HAZ and WAZ scores show a steady decline with increasing child age, reflecting age-related growth vulnerabilities. Children born with smaller sizes exhibit substantial growth deficits, with a decline of 0.32 SD in HAZs and 0.38 SD in WAZs. Similarly, a history of diarrhea is linked to significant reductions in both HAZ and WAZ scores.

Maternal education contributes positively to growth outcomes, with a significant 0.017 SD improvement in HAZs and a 0.02 SD improvement in WAZs per additional year of education. Access to healthcare is measured through prenatal checkups, hospital deliveries, cesarean deliveries, and postnatal checkups, along with maternal age and height, also yields significant positive effects on both HAZ and WAZ scores. However, larger household size, higher birth orders, and maternal smoking are negatively associated with both HAZ and WAZ scores, highlighting the compounded vulnerabilities in resource-constrained households. Furthermore, provincial and regional differences are evident, with children in Sindh, Baluchistan, and the former Federally Administered Tribal Areas (FATA) exhibiting significantly lower HAZ and WAZ scores than those in Punjab (reference province). In contrast, children in Islamabad Capital Territory (ICT) and Khyber Pakhtunkhwa (KP) perform better. Finally, the alternative model specifications presented in Columns 4 and 5 are fully consistent with the OLS estimates in both direction and statistical significance.

Table 4 presents the results of Blinder–Oaxaca decomposition, detailing the contributions of different components to the mean HAZ and WAZ gaps between non-poor and poor households. It is pivotal to clarify the differences between endowment effects and coefficient effects as endowment effects showcases differences in characteristics (structural factors), while the coefficient effects reflects disparities in returns to those characteristics (behavioral or systemic factors). This distinction is made to clarify whether observed inequalities arise from unequal resources distribution or from differential treatment between non-poor and poor groups. Specifically, mean HAZ gap of 0.74 indicates that HAZ scores for poor households were three-quarters of a standard deviation (SD) lower than those for non-poor households. Of this gap, 62% (0.46/0.74) is attributable to the endowment effect (structural factors), reflecting differences in the magnitude of covariates, whereas 16% (0.12/0.74) is due to the coefficient effect, representing behavioral disparities in how these covariates translate into outcomes. The interaction term accounts for the remaining 22% of the gap.

**Table 4:** Decomposing predicted changes in child growth outcomes, non-poor and poor.

**Overall**	**HAZ score**			**WAZ score**		
Non-poor	−1.307^***^			−0.917^***^		
	(0.026)			(0.020)		
Poor	−2.056^***^			−1.423^***^		
	(0.032)			(0.023)		
Difference	0.749^***^			0.506^***^		
	(0.042)			(0.031)		
Endowments	0.466^***^			0.275^***^		
	(0.057)			(0.041)		
Coefficients	0.124^*^			0.023		
	(0.066)			(0.049)		
Interaction	0.160^**^			0.208^***^		
	(0.076)			(0.056)		
31.5-197.35,15.8**Decomposition effects**						
**Variables**	**Endowments**	**Coefficients**	**Interaction**	**Endowments**	**Coefficients**	**Interaction**
Female child	−0.002	−0.033	0.001	−0.000	0.006	−0.000
	(0.002)	(0.038)	(0.001)	(0.001)	(0.028)	(0.001)
Child's age	0.006	0.186^**^	−0.001	0.003	0.064	−0.000
	(0.013)	(0.075)	(0.003)	(0.006)	(0.055)	(0.001)
Small size at birth	0.012^***^	0.020	−0.003	0.011^***^	−0.007	0.001
	(0.004)	(0.019)	(0.003)	(0.004)	(0.014)	(0.002)
Diarrhea	0.003	−0.004	0.000	0.005^*^	0.026^*^	−0.002
	(0.002)	(0.021)	(0.002)	(0.003)	(0.015)	(0.002)
Prenatal care	0.031^**^	−0.006	−0.002	0.026^**^	0.013	0.005
	(0.014)	(0.049)	(0.019)	(0.010)	(0.036)	(0.014)
Hospital delivery	0.017	0.050	0.036	0.019	0.023	0.017
	(0.021)	(0.040)	(0.029)	(0.016)	(0.030)	(0.021)
Cesarean delivery	0.055^***^	−0.015	−0.028	0.062^***^	−0.020^**^	−0.038^**^
	(0.019)	(0.011)	(0.021)	(0.014)	(0.008)	(0.015)
Postnatal care	0.001	−0.018	−0.007	−0.000	−0.000	−0.000
	(0.006)	(0.019)	(0.007)	(0.004)	(0.014)	(0.005)
Birth order number	0.054^***^	0.217^**^	−0.046^**^	0.010	0.056	−0.012
	(0.016)	(0.098)	(0.021)	(0.011)	(0.073)	(0.015)
Women's age	−0.000	−0.417	0.000	−0.000	−0.095	0.000
	(0.005)	(0.273)	(0.002)	(0.002)	(0.202)	(0.000)
Respondent's height	0.045^***^	1.152	0.007	0.027^***^	0.781	0.005
	(0.008)	(1.016)	(0.007)	(0.005)	(0.749)	(0.005)
Maternal education	0.118^**^	−0.011	−0.031	0.098^***^	0.001	0.002
	(0.048)	(0.020)	(0.055)	(0.035)	(0.015)	(0.041)
Husband education	0.019	0.057	0.047	0.018	0.051	0.042
	(0.027)	(0.043)	(0.035)	(0.020)	(0.031)	(0.026)
Mother smoke	0.008^**^	0.022^**^	−0.006^*^	−0.002	0.006	−0.002
	(0.004)	(0.009)	(0.003)	(0.002)	(0.007)	(0.002)
No water facility	0.009	0.015	−0.009	0.017^***^	0.023	−0.014
	(0.008)	(0.021)	(0.013)	(0.006)	(0.016)	(0.010)
No toilet facility	0.011	−0.139^***^	0.128^***^	0.001	−0.086^**^	0.079^**^
	(0.018)	(0.051)	(0.047)	(0.013)	(0.038)	(0.035)
Member of household	0.001	−0.154^**^	0.003	−0.000	−0.062	0.001
	(0.001)	(0.075)	(0.002)	(0.001)	(0.056)	(0.001)
BISP	0.000	0.005	−0.002	0.002	0.001	−0.000
	(0.004)	(0.012)	(0.005)	(0.003)	(0.009)	(0.004)
Category-Sindh	0.011^*^	0.028	−0.002	0.010^*^	0.027	−0.002
	(0.006)	(0.025)	(0.002)	(0.006)	(0.018)	(0.002)
Category-KP	−0.004	−0.000	0.000	−0.006^**^	−0.018	0.002
	(0.003)	(0.023)	(0.003)	(0.003)	(0.017)	(0.002)
Category-Baluchistan	0.030^***^	0.031	−0.010	0.007^*^	−0.035^**^	0.011^**^
	(0.007)	(0.020)	(0.006)	(0.004)	(0.015)	(0.005)
Category-GB	−0.008^**^	−0.035^**^	0.011^**^	−0.025^***^	−0.036^***^	0.012^***^
	(0.004)	(0.016)	(0.006)	(0.006)	(0.012)	(0.004)
Category-ICT	0.009	0.002	0.007	0.010	−0.001	−0.005
	(0.019)	(0.004)	(0.020)	(0.014)	(0.003)	(0.015)
Category-AJK	−0.009	0.006	0.008	0.001	0.002	0.002
	(0.008)	(0.007)	(0.009)	(0.006)	(0.005)	(0.007)
Category-FATA	0.030^***^	−0.035	0.030	−0.014^*^	−0.048^***^	0.041^***^
	(0.010)	(0.022)	(0.018)	(0.007)	(0.016)	(0.014)
Urban	−0.005	0.018	0.035	−0.014	0.023	0.046
	(0.033)	(0.020)	(0.040)	(0.024)	(0.015)	(0.029)
Year = 2018	0.024^*^	−0.012	−0.005	0.008	0.046	0.018
	(0.013)	(0.041)	(0.016)	(0.009)	(0.030)	(0.012)

^***^p < 0.01, ^**^p < 0.05, ^*^p < 0.1.

Similarly, the mean WAZ gap between the groups was 0.50, with 54% (0.27/0.50) explained by the endowment effect and only 4% (0.02/0.50) explained by the coefficient effect. This means that structural factors account for 54%, whereas behavioral characteristics explain only 4%. The interaction term contributes to the remaining 42% of the gap. These findings emphasize the importance of resource disparities (endowments) in explaining nutritional inequalities while highlighting the need to address structural differences (coefficients) to reduce persistent gaps. This analysis underscores the importance of tailored policy interventions that target both resource allocation and systemic inequities to improve child nutritional outcomes.

The contribution of both components, endowments and coefficients, to the HAZ gap was statistically significant. The endowment component represents the potential improvement in the HAZ scores of children from poor households if they have the same level of predictors as their non-poor counterparts. The coefficient component, on the other hand, reflects the change in HAZ scores for children from poor households if they were to benefit from the same effects of predictors as non-poor households, given their current levels of predictors.

Among the covariates, maternal education emerged as the most significant contributor to the endowment effect, accounting for 25% of the gap. This was followed by cesarean delivery and birth order (12% each), maternal height (9%), prenatal care (6%), regional disparities in Baluchistan and FATA (6%), and the year 2018 (5%). These variables collectively account for a significant proportion of the gap due to the endowment effect. Variables with negative contributions, such as those from Gilgit-Baltistan (GB), mitigated the HAZ gap between poor and non-poor households.

## 4 Discussion

This study examines the persistent cycle of nutritional inequality among children in Pakistan, classified by household asset index into poor and non-poor households. It investigates the key determinants contributing to these disparities. Using parametric and semi-parametric methods in conjunction with the Blinder–Oaxaca decomposition, the analysis unpacked the observed differences in HAZ and WAZ scores into endowment (characteristics) and coefficient (returns to characteristics) components. This empirical method provides a nuanced understanding of how disparities in child growth outcomes are determined by both differences in socio-economic, demographic, and regional characteristics, as well as differences in the effects of these characteristics across groups. Accordingly, a significant portion of the gap is explained by differences in demographic and socioeconomic factors, such as small size at birth, diarrhea incidence, birth order, a mother who smokes, and no access to water facilities, for which the percentage of poor individuals is greater than that of non-poor individuals as well as prenatal checkups, cesarean delivery, parental height, and parental education for which the percentage of non-poor individuals is greater than that of poor individuals.

The findings of this study corroborate and enhance the data presented in the previous studies. Small size at birth emerged as a predominant factor driving nutritional inequality, indicating that poverty directly limits access to adequate nutrition (24, 25). A significant negative relationship exists between the socioeconomic status of a household and low birth weight, manifesting how poverty may retard access to adequate nutrition, thus extending the nutritional gap (26). Martinson (24) reveals that socioeconomic status associated with low birth weight was more prevalent in the United States than in the United Kingdom, Canada, and Australia, suggesting that income disparity and limited social support systems further exacerbate the nutritional gap. In line with previous studies (27, 28), this research further establishes that maternal smoking and elevated birth order are adversely correlated with children's nutritional outcomes, hence heightening the risk of malnutrition and obesity.

Additionally, the substantial influence of maternal education is positively associated with improved nutritional outcomes, demonstrating the intergenerational rewards of investment in educational attainment (21, 22, 29–31). On the other hand, cesarean deliveries prevent complications, thereby indirectly supporting improved nutritional outcomes. This is supported by studies providing a significant positive association between cesarean delivery and child nutritional outcomes. However, the current analysis shows that access to prenatal check-ups, cesarean delivery, and educational attainment remains limited among poorer groups. Thus, improved healthcare infrastructure, targeted maternal education, and equitable resource distribution are critical for alleviating the nutritional gap and promoting health system equality.

The study also presents significant nutritional inequalities at the regional level. For example, compared with their urban counterparts, children in rural areas have poorer nutritional indicators, primarily due to limited access to healthcare, clean drinking water, and sanitation facilities. These findings suggest that poverty not only restrain material sources but also blocks the capacity of the families to convert the available resources into enhanced child health outcomes.

This study offers valuable insights and proposes practical intervention programs for decision-makers. Since prenatal checkups and maternal education account for most of the explained inequality, integrating nutrition counseling into antenatal programs could yield significant equity gains. Similarly, closing the endowment gaps requires an educational support program, job opportunities, and accessibility to health services for all. At the same time, addressing the unexplained component of the nutritional gap involves tackling structural inequalities through policy intervention programs such as social protection schemes for needy households, an accessible healthcare system, and launching initiatives that reduce discrimination and enhance efficiencies in resource allocation.

Despite the robust methodology, this study has also numerous limitations. First, this study uses a cross-sectional dataset, which often limits the ability to establish causality between socioeconomic determinants and child nutritional outcomes. Second, several potentially influential variables, such as household food security, maternal mental health, and intra-household resource allocation, are either absent or insufficiently detailed in the PDHS dataset. Third, recall bias and reporting inaccuracies, particularly in indicators such as birth size, could affect the precision of the estimates. Lastly, unobserved cultural, behavioral, and environmental factors, which may differ significantly across regions and socioeconomic groups, remain part of the unexplained component. However, the study uses year dummies, which, to a greater extent, capture these unobserved differences.

## Data Availability

Publicly available datasets were analyzed in this study. This data can be found at: https://microdata.worldbank.org/index.php/catalog/3411/related-materials.
